# Direct and Secondary Transfer of Touch DNA on a Credit Card: Evidence Evaluation Given Activity Level Propositions and Application of Bayesian Networks

**DOI:** 10.3390/genes14050996

**Published:** 2023-04-27

**Authors:** Martina Onofri, Cristina Altomare, Simona Severini, Federica Tommolini, Massimo Lancia, Luigi Carlini, Cristiana Gambelunghe, Eugenia Carnevali

**Affiliations:** 1 Section of Legal Medicine, Department of Medicine and Surgery, University of Perugia, 06123 Perugia, Italy; cristalt97@gmail.com (C.A.); massimo.lancia@unipg.it (M.L.); luigi.carlini@unipg.it (L.C.); cristiana.gambelunghe@unipg.it (C.G.); 2 Section of Legal Medicine, Department of Medicine and Surgery, Santa Maria Hospital, University of Perugia, 05100 Terni, Italy; s.severini@aospterni.it (S.S.); f.tommolini@aospterni.it (F.T.); e.carnevali@aospterni.it (E.C.)

**Keywords:** touch DNA, direct transfer, secondary transfer, mixtures, background DNA, evidence evaluation, likelihood ratio, activity level propositions, Bayesian Networks

## Abstract

In a judiciary setting, questions regarding the mechanisms of transfer, persistence, and recovery of DNA are increasingly more common. The forensic expert is now asked to evaluate the strength of DNA trace evidence at activity level, thus assessing if a trace, given its qualitative and quantitative features, could be the result of an alleged activity. The present study is the reproduction of a real-life casework scenario of illicit credit card use by a co-worker (POI) of its owner (O). After assessing the shedding propensity of the participants, differences in DNA traces’ qualitative and quantitative characteristics, given scenarios of primary and secondary transfer of touch DNA on a credit card, a non-porous plastic support, were investigated. A case-specific Bayesian Network to aid statistical evaluation was created and discrete observations, meaning the presence/absence of POI as a major contributor in both traces from direct and secondary transfer, were used to inform the probabilities of disputed activity events. Likelihood Ratios at activity level (LRα) were calculated for each possible outcome resulting from the DNA analysis. In instances where only POI and POI plus an unknown individual are retrieved, the values obtained show moderate to low support in favour of the prosecution proposition.

## 1. Introduction

Forensic genetics has been considered the gold standard for investigative and judiciary purposes to assess whose DNA has been recovered from a crime scene and to aid the Court in identifying the individuals who may have taken part in the dynamic of the criminal event [1]. However, the identification of the donor of a biological trace in and of itself is not sufficient anymore. Forensic experts are increasingly required to aid in the understanding of the relevance of said biological trace for the criminal case in question. Questions concerning the timing and mechanisms of deposition, and the effect that other variables, such as recovery and persistence, contamination, and presence of background DNA, may have on findings are ever more common. This is particularly pertinent in the case of analysis and evaluation of trace DNA, meaning scarce quantities of DNA, usually a mixture, not attributed to a specific body fluid [2,3].

Since hands are the most prominent means of interaction with objects and surroundings (not necessarily in a criminal offence), these considerations also apply to touch DNA, meaning biological material released from the skin, derived from shed keratinocytes, nucleated cells from other body parts, secretions, and cell-free DNA [1,2,3,4,5,6,7,8]. The last is particularly informative and yields a higher amount of DNA than other components; however, its origin is not yet clear [5,7,9]. The amount of touch DNA that individuals may deposit, known as shedder status, varies greatly due to intra- and inter-individual variables. Body location, age, sex, skin conditions, activity performed, type and length of contact, and the surface of contact are all factors that increase variation in terms of shedding propensity [4,10,11,12,13,14,15,16,17].

Thanks to the increase in sensitivity of the current forensic genetics techniques, it is now possible to recover and obtain better DNA profiles from minute quantities of biological material, such as trace and touch DNA. Despite the obvious advantage of being able to infer more robust results and relevant information from limited amounts of DNA, this improvement led to the generation of more complex DNA profiles, often multi-contributors, with increased detection of background DNA [14,15,18,19,20,21,22,23,24], contamination events, and DNA profiles not resulting from a direct transfer. In particular, DNA transfer, especially secondary or higher level through one or more intermediate vectors, is a well-recognised phenomenon that further complicates DNA analysis results’ interpretation [4,6,10,16,22,25,26,27,28,29,30,31].

Specifically, in a judiciary setting, such considerations moved the evaluation of the DNA evidence to a further step in the hierarchy of propositions. With hierarchy of propositions, we define a set of broad categories at which the evidence can be evaluated depending on the characteristics that are taken into consideration, the information available, and the propositions that are being formulated [32,33]. In particular, the evaluative framework was moved from a (sub)source level, where the question “Whose DNA is this?” is answered [34], to the activity level, where the source of the DNA is not disputed, but the mechanism of transfer and the influence of other case-specific factors are [26,27,35,36,37]. Assuming that the suspect is the origin of a biological trace, it is essential to explore how the DNA of the suspect got to the crime scene, either via a criminal activity, secondary or higher degree transfer, or a legitimate activity [30,38]. For this kind of estimation, the experts assess the strength of the evidence given competing alternative hypotheses (Hp and Hd) regarding the activity being disputed. Possible mechanisms and instances of DNA transfer and their relative likelihood have to be presented, in light of the Influence that other case-specific factors may have. Therefore, it’s important to properly evaluate the case circumstances and to assess if a trace, given its qualitative and quantitative features, could be the result of a primary or further level of transfer [6,23,31,35,39,40,41,42,43]. This approach is termed evaluative reporting, and it has to be objective and based on case-relevant information [35,36,37]. It is recommended that the expert opinion is informed by empirical and experimental data, present in the literature or produced in-house. Given the high number of case-specific relevant variables and of data regarding the activities that may have led to the deposition and transfer of DNA, the experts are increasingly relying on the use of Bayesian Networks (BN) [38,44,45,46,47,48]. BNs are graphical representations of the case that include all the relevant variables and display their dependency relationships and interactions. Taking into consideration all the important and pertinent elements to the case, BNs allow making complex statistical evaluations of the DNA analysis results, based on every possible combination of events and on which disputed activity is assumed as true (according to either Hp or Hd) [22,31,44,45,49,50,51].

As mentioned above, Bayesian Network construction and more generally DNA evidence evaluation at the activity level have to be informed by experimental data. When probabilistic observations on relevant case-specific variables are not available from previously published, peer-reviewed studies, the expert must either adapt existing data, if not specific, or produce in-house results from ad hoc experiments. The present study is an example of a touch DNA evidence evaluation given activity level propositions regarding the direct manipulation of a credit card and, albeit this assessment was not required for judiciary purposes, the case scenario which prompted such an investigation.

## 2. Materials and Methods

### 2.1. Case Circumstances

For a case of alleged fraudulent use of a credit card, our laboratory was commissioned to perform DNA analysis and evaluation only at the sub-source level. However, the case circumstances prompted the experimental reproduction of the case and the investigation of touch DNA transfer mechanisms and trace evaluation given activity level propositions.

The credit card owner (O) works in an office shared with another individual. While on sick leave, she noticed unauthorised withdrawals from her bank account. The card linked to said bank account was left in her private locker in the office while she was on leave. Given circumstantial evidence, the person suspected of having unduly used the card was her co-worker (POI). POI stated that he never used O’s card and that he never handled it. He stated, however, that O was known for not storing the card in a personal and safe place. She often left it on her and other co-workers’ desks, including POI’s. When getting back to the office, O did not touch the card again, only checking for its presence in her locker. It is assumed that no one else touched the card since it got back into the owner’s possession. The DNA traces found on O’s card turned out to be compatible with a mixture of O, POI, and an unknown individual, to which POI was the second most prominent contributor. Relative mixture proportions were calculated with EuroForMix v. 4.0.1 (quantitative LR model) and returned values of 0.5 for O, 0.4 for POI, and 0.1 for the unknown individual.

We decided it was worthwhile replicating and studying the event, to investigate the differences (if existing) in DNA traces’ characteristics for primary deposition and secondary transfer of touch DNA on a credit card, a non-porous plastic support. Particularly for the secondary transfer, additional interest arose from the social setting, meaning that the individuals shared the same working space, thus possibly increasing the complexity of the results interpretation [28,36,50].

The study was approved by the Ethics Committee of the University of Perugia (protocol code 92184). To mirror the co-working conditions of this scenario, laboratory staff volunteered for the study and signed informed consent to the analysis of the deposited DNA traces and to the use of their genotypes, present in the exclusion database of the laboratory, for reference purposes. A total of four participants were included in the study. However, to minimize the possibility of contamination events, the sample collection and laboratory processing phase were carried out by personnel who did not participate in the study, and, during this process, participants were not allowed in the laboratory spaces, after their careful sterilization.

The experimental set-up of the study consisted of four phases.

### 2.2. Phase I—Shedder Status

Since shedder status influences transfer, persistence, and recovery of DNA, phase I of the experiment consisted of the shedder status assessment for all participants (coded from A to D). Participants were first asked to wash their hands with antibacterial soap and then dry them with a sterile, personal towel. Immediately after handwashing, participants were asked to hold, with both hands and for 30 s, a 15 mL falcon tube that had been previously sterilized with UV lights for 1 h. The falcon tubes were immediately sampled and labelled with the code of the participant and the time point of the sampling, *t*0. The volunteers were asked to repeat the experiment at three additional time points after handwashing, 1 h, 2 h, and 3 h, respectively. These samples were labelled with the code of the participant and the relative time point, meaning *t*1, *t*2, and *t*3. During the time elapsed between the handwashing and the experiment at *t*3, participants were not given any particular imposition but were instructed to carry out routine daily activities; however, hand washing was not allowed.

Four shedding samples per participant were collected, for a total of 16 samples. The focus of this phase was not strictly the investigation of shedding status variability among individuals. It was, rather, the informed pairing of participants in phases II and III, to obtain variability in the pairings while also maintaining even differences among couples. The aim is to preserve similar experimental conditions with different individuals. Additionally, information on the participants’ shedder status was collected to better interpret phases II and III results.

### 2.3. Phase II—Direct Transfer

Pairings of participants were established based on the results of phase I. In phase II, the direct transfer scenario was replicated. Participants were asked to provide one or more frequently used credit cards, for a total of 11 cards. The card, whose owner was indicated with the code “O”, was then manipulated, without gloves, by the counterpart in the pairing, labelled as “POI”. The card was handled for 30 s by POI, who then simulated an ATM cash withdrawal.

To replicate realistic case-work conditions, the cards were not sterilized, nor were the participants asked to wash their hands. The cards were then immediately sampled, and the samples were assigned the code from D1 to D11 (“D” standing for Direct transfer).

### 2.4. Phase III—Secondary Transfer

After the sampling in phase II, the cards were sterilised by means of UV lights, 30 min per side, to remove any possible DNA trace from POI that may have persisted on the cards. The owners were then asked to use them as they regularly do for a month. After this period, phase III was carried out: the pairings from phase II were maintained and the same number of cards was used (11 cards). In this phase, the secondary transfer scenario was investigated, meaning that the owner’s (O) card was placed and moved around the surface of POI’s desk, applying slight pressure, for 30 s. To conform to realistic experimental conditions, the desks’ surfaces and the cards were not sterilized. However, the desks are personal to the participants and before the card was placed upon them, the POIs touched the surfaces with their bare hands and forearms, to replicate a “uniform” use of the desk among participants. The cards were immediately sampled, and the samples were given codes ranging from S1 to S11 (“S” standing for Secondary transfer).

### 2.5. Phase IV—Laboratory Processing and Statistical Analysis

Through all the experimental phases, the items (falcon tubes and cards) were sampled by using the tape lifting technique. Despite a more difficult interpretation of results, this recovery method has yielded a higher amount of DNA than other techniques [52,53,54,55]. All samples were extracted employing the phenol–chloroform organic method [56,57], and the pellet of each sample was resuspended in 30 μL of sterile water. The DNA extracts were quantified using the PowerQuant^®^ System (Promega, Madison, WI, USA) [58,59]. As described in Section 3, quantification results were quite low; therefore, samples were later amplified undiluted as is, and no DNA concentration threshold was applied to further process the samples, given the explorative approach of this study. PCR amplification was carried out using the PowerPlex^®^ ESX17 Fast kit (Promega, Madison, WI, USA), as per the manufacturer’s instructions [60,61]; given the nature of the study, no modifications for low template DNA amplification were applied. Capillary electrophoresis followed by means of the Applied Biosystems^TM^ SeqStudio^TM^ Genetic Analyzer (Thermo Fisher Scientific, Waltham, MA, USA) with the following conditions: injection time, 7 s; injection voltage, 1200 V; run time, 1440 s; run voltage, 9000 V. Results were analysed using the software GeneMapper^®^ ID-X v 1.6 (Thermo Fisher Scientific, Waltham, MA, USA). Analytical threshold for alleles (AT) was set at 50 RFU. As described in Section 3, further statistical analyses and calculation of LR at the sub-source level (LRϕ) were performed using EuroForMix v 4.0.1. Reference profiles of the participants were already available in the laboratory exclusion database. The LR at activity level (LRα) was calculated manually and results were confirmed by using the software Hugin Expert. The plots in this paper were created by using the R package ggplot2.

## 3. Results

### 3.1. Phase I—Shedder Status

**Figure 1:**
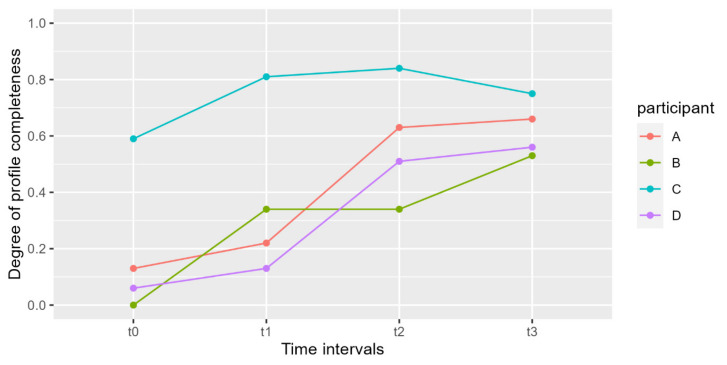
Graphical representation of the increase in profile completeness degree of touch DNA samples deposited by the four participants at four different time points after handwashing: *t*0 (immediately after), *t*1 (1 h), *t*2 (2 h), and *t*3 (3 h). Participants were asked to hold, for 30 s and with both hands, a 15 mL falcon tube.

All data regarding the results of phase I are reported in Table 1; DNA quantity and profile completeness of the donor of the trace were investigated. The correlation between profile completeness for each participant and time elapsed from hand washing is plotted in Figure 1.

Samples showed a concentration ranging from 0.0005 to 0.0270 ng/μL, for a total of 0.015 to 0.810 ng in 30 μL. As expected, the samples collected at *t*0 yielded the lowest DNA amounts. For the following time points an increase in DNA amounts, consistent among participants, was observed. These results are mirrored by the value of profile completeness, calculated on the proportion of alleles present in the sample that match the profile of the participant.

The overall lower results are reported for participant B; however, it is possible to observe only slight differences with individuals A and D. Only participant C shows a distinct trend, both in terms of quantitation and profile completeness, yielding the highest values. Participant D sheds, on average, twice as much DNA as participant B (Table 1). Taking into consideration previously published works and our results, shedder categories were established based on profile completeness, keeping in mind quantitation results. Additionally, given our observation that 3 out of 4 individuals displayed intermediate values, we established three shedding categories: Poor shedder (profile completeness degree between 0 and 0.3), Intermediate shedder (profile completeness degree between 0.31 and 0.60), and Good shedder (profile completeness degree between 0.61 and 1). Participant C fell into the Good shedder category and participants A and D were in the Intermediate. Given the thresholds we established, participant B was classified as a Poor shedder; however, as mentioned above, the results obtained did not differ much from those observed for participants A and D (Table 1, Figure 1).

Based on these results, couples were created pairing Poor with Intermediate shedders and Intermediate shedders with Good shedders, to create more homogenous results while maintaining a margin of variability.

### 3.2. Phases II and III—Direct and Secondary Transfer

**Figure 2:**
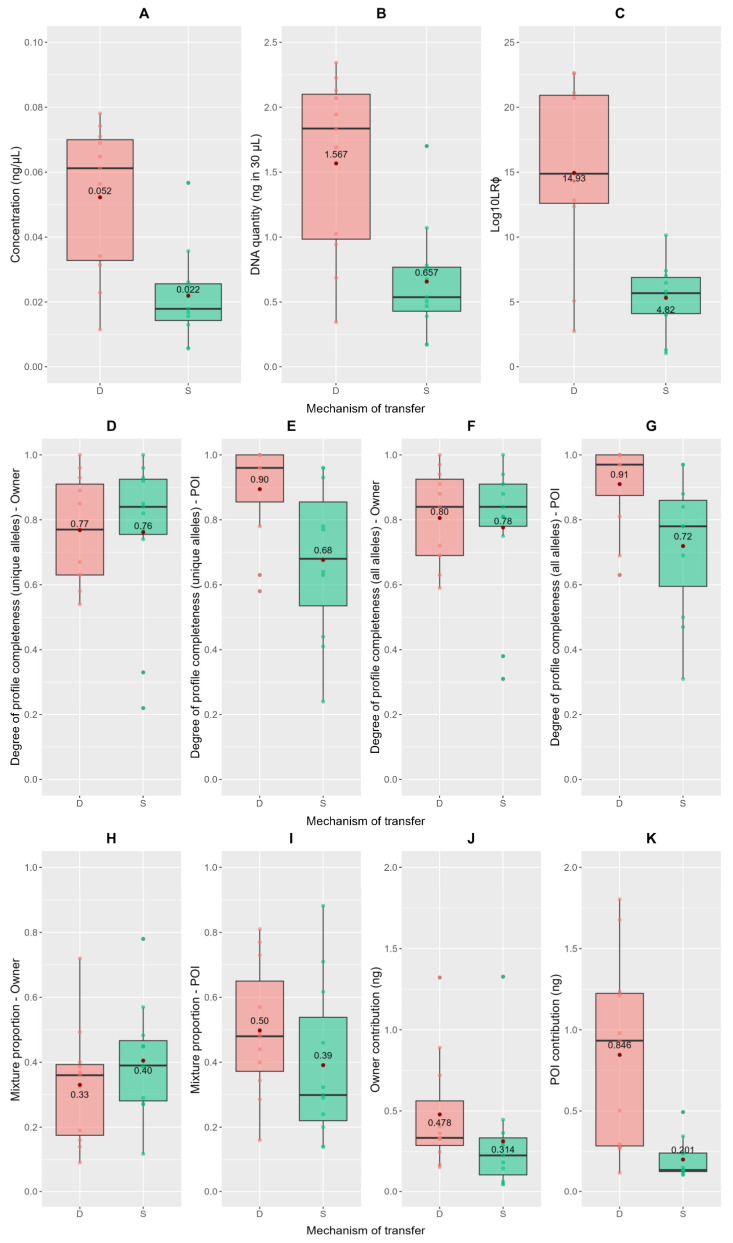
Boxplots showing the DNA trace attributes investigated. (**A**,**B**) DNA deposited, as concentration (ng/μL) and DNA quantity (ng in 30 μL); (**C**) logarithmic value of the LR at sub-source level (log10LRϕ); (**D**–**G**) degree of profile completeness, for both O and POI, calculated taking into consideration both the proportion of unique alleles (**D**,**E**) and the proportion of all possible alleles (**F**,**G**) present in the trace; (**H**,**I**) mixture proportion, for both O and POI, calculated by EuroForMix v 4.0.1; (**J**,**K**) O and POI’s contribution to the trace in terms of DNA quantity (ng), calculated by multiplying O and POI relative mixture proportion with the DNA quantity (ng in 30 μL) of the trace. Values observed for these characteristics are discriminated between direct and secondary transfer mechanisms, indicated as “D” and “S”, respectively. Mean values are marked as red dots and their numerical value is reported in the boxplots.

Results were initially evaluated taking into consideration quantitation data and O/POI’s profile completeness. Results are reported in Table 2 and the differences observed between direct and secondary transfer are shown in Figure 2, along with their mean values.

DNA traces from the primary transfer scenario show a DNA quantity ranging from 0.345 ng to 2.343 ng (in 30 μL), with an average of 1.6 ng. These results are around double the values of DNA quantities retrieved in the secondary transfer scenario (from 0.171 to 1.7 ng, with an average value of 0.657 ng in 30 μL) and they are consistent with previous findings [17,22,62,63] (Figure 2A,B).

Profile completeness was calculated with a double approach. For both O and POI, it was initially calculated as the number of unique alleles (alleles not shared by O and POI) present in the trace divided by the number of possible unique alleles (of either O or POI) [42,64,65,66,67] (Figure 2D,E). Profile completeness was then calculated based on all the possible alleles that could be observed in the reference profiles, including the alleles shared by O and POI (Figure 2F,G). For both calculation methods, the profile completeness of the owner (O) is consistent between primary and secondary transfer events, as expected with consistent use of the card in both instances. Instead, the profile completeness of POI drops from a mean value of 0.90 in the primary transfer to 0.68 in the secondary transfer event for the “unique alleles” method, while dropping from a value of 0.91 to 0.72 for the “all alleles” calculation approach.

Further statistical analysis was performed by using EuroForMix v 4.0.1 [68], results are reported in Table 3 and Figure 2. For each sample, the Likelihood Ratio at sub-source level (LRϕ) was calculated using the continuous (quantitative) model with the following parameters: AT set at 50 RFU; FST correction set at 0; PrC set at 0.05; lambda set at 0.01. Model options for degradation, forward and backward stutters, and number of contributors were chosen for each DNA trace based on the optimal adjLoglik value recommended by EuroForMix’s “automatic model search option”.

As further discussed in Section 3.4, a discriminatory value of 10^6^ for LR at sub-source level was taken into consideration. In 82% (9 out of 11) of primary transfer samples, an LRϕ greater than 10^6^ was obtained, thus sufficient to agree to the identity of POI as a contributor to the trace. In the secondary transfer scenario, only 36% of the samples (4 out of 11) yielded a log10LRϕ greater than 6 (Table 3). As shown in Figure 2C, mean values of log10LRϕ vary greatly between transfer mechanisms, dropping from a value of 14.93 for direct transfer to 4.62 for secondary transfer. In this case, a low LR (log10LRϕ < 6) corresponded to a very low DNA quantity yield.

Mixture proportion values for O, POI, and unknown (for samples with three contributors) were calculated by EuroForMix under Hp and are reported in Table 3 and Figure 2H,I. O and POI’s contribution to the trace in terms of DNA quantity (ng) was calculated by multiplying O and POI’s relative mixture proportion with the DNA quantity (ng in 30 μL) of the trace (Table 3, Figure 2J,K). The owner’s contribution is expected to be quantitatively the same for both direct and secondary transfer; however, a slight decrease in DNA amounts is observed. O’s contribution drops from a mean value of 0.478 ng in direct transfer to 0.314 ng. A possible explanation is discussed in Section 4.2.

As can be observed (Table 3, Figure 2I,K), in the direct transfer simulations, the last user (POI) contributes generally more to the make-up of the trace. In terms of mixture proportion, POI’s contribution drops slightly, from a mean of 0.5 in the primary transfer to a value of 0.39 in the secondary transfer (Figure 2I). At the same time, the mean value of DNA quantity (ng) deposited by POI in the direct transfer (0.846 ng) is around four times greater than the mean value for the secondary transfer experiment (0.201 ng), given that POI’s presence is the result of an indirect contact through a surface (Figure 2K). The correlation between contribution and DNA amount is to be expected since the mixture proportion is calculated based on the observed alleles matching the individual’s reference genotype and their respective peak height (in RFU), which is, in turn, proportional to the trace’s DNA amount deposited by the individual [67]. However, for the secondary transfer scenario, the decrease of POI’s contribution in terms of mixture proportion lead to an increase in O’s mixture proportion value, albeit corresponding to a slightly lower DNA yield. Additionally, the results of O and POI’s contributions to the trace are broadly reflective of the results observed for the profile completeness values.

Ultimately, POI was a major contributor, both in terms of mixture proportion and DNA amount contributed to the trace, in 64% (7 out of 11) of the primary transfer samples and only in 36% (4 out of 11) of the secondary transfer events.

Student’s two-way *t*-tests were used to compare variables expected to differ between the mode of transfer (direct and secondary): DNA quantity (ng in 30 μL), log10LRϕ, and parameters referring to POI’s contribution to the trace, meaning profile completeness, mixture proportion, and DNA amount contribution. Two variables, log10LRϕ (*p*-value of 0.0019) and POI contribution in terms of DNA amount (*p*-value of 0.0057), were chosen as the criteria for the calculation of direct and secondary transfer mechanisms’ probabilities, as will be discussed in Section 3.4.

### 3.3. Background DNA

Background DNA is defined as the presence of alleles, not observed in the prevalent DNA, due to unknown sources [36]. As can be observed in Table 3, 19 out of 22 total samples presented 3 contributors, therefore we estimated that background DNA was present in 86.4% of the samples. The third, unknown contributor had a higher relative contribution to the mixture in the secondary transfer rather than in the direct transfer. In one sample (S8), the third contributors’ value was higher than POI’s contribution, approximating the value calculated for the owner.

In our case, given that the objects involved in the study (cards and personal desks) were not sterilized, nor had the participants washed their hands before handling the cards, it is not possible to determine the source of background DNA. Therefore, for our purposes, only a qualitative assessment of the presence or absence of background DNA is performed. This assessment was, however, possible for samples from phase I: 5 out of 16 samples presented a second contributor. In this case, the non-prevalent DNA must have originated from the hands of the participant [14,43]. This data, albeit informative, was not included in the probabilistic calculations described below.

### 3.4. Constructing a Bayesian Network

A *Bayesian Network* (BN) is a graphical representation of complex probability calculations and is becoming a commonly used tool in evaluating the strength of the evidence under different information and assumptions [33,38,44,45,46,47,48]. BNs are highly customisable and can be constructed reflecting case-specific scenarios: in this study, DNA results are evaluated under competing propositions regarding DNA deposition activities. Therefore, based on the circumstances of the case in the study, the relevant information was identified as follows:

- O and POI share the office workspace;
- O often left her credit card on POI and other co-workers’ desks;
- If POI did not use the card, someone else did (evidence given by the bank withdrawal).

In addition, it is assumed, by both the prosecution and the defence, that POI is the origin of the DNA retrieved from the credit card. Since what is disputed is not the identification of POI as a donor to the DNA trace but how the DNA got on the card, the evidence is evaluated given the following alternative propositions:

**Hp:**  *POI used O’s card and made the cash withdrawals (primary transfer);*

**Hd:**  *POI did not use the card, an unknown individual did, and the presence of POI as a contributor to the trace is due to coworking (secondary transfer).*

Hd supports legitimate reasons for POI’s DNA to be on the card through previous innocent transfer.

**Figure 3:**
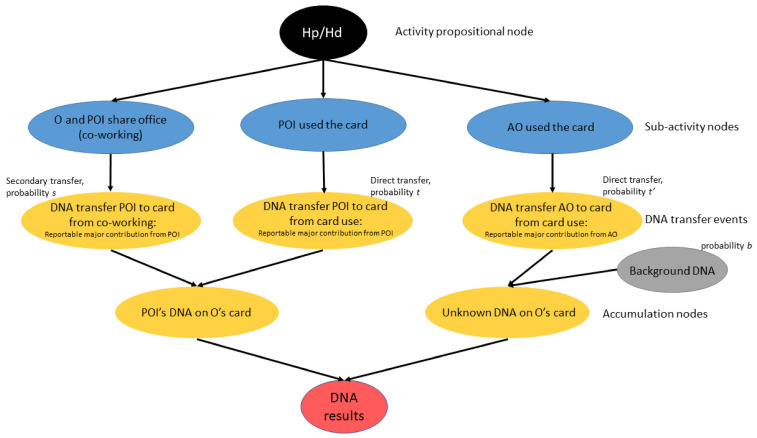
Design of the Bayesian Network here constructed. Sub-activity nodes, DNA transfer events, and accumulation nodes are all described based on possible disputed DNA transfer mechanisms. The “Background DNA” root node is a parental node only to the accumulation node “UNK’s DNA on O’s card”. *t*, *t’*, *s*, and *b* probabilities were informed by experimentation. To infer the probability of DNA transfer for both direct and secondary transfer mechanisms, two criteria were applied. The observation of a reportable profile (log10LRϕ > 6) has been labelled as the 1st level criterion. The 2nd level criterion is POI being the major contributor in terms of DNA amounts.

Starting from these two alternative competing propositions, the Bayesian Network (BN) was constructed according to the guidelines provided by various works [36,43,45,48,49,50,65]. The construction of the BN was case-specific, meaning that it was tailored to the case-relevant information and the mechanisms of transfer we investigated. Its design is shown in Figure 3. The first node, defined as the *main proposition node*, reflects the hypothesis of the prosecution and defence, which are stated above. The nodes that directly depart from the main proposition node are known as *activity nodes* and they describe the activity that is being disputed, in our case the mechanism of transfer that could have led to POI’s DNA transfer to the card:

- POI and O share the workspace;
- POI used the card (without gloves);
- An unknown alternative offender (AO) used the card (without gloves).

Below activity nodes, *finding nodes* are placed and they describe the DNA transfer events, meaning the probability of recovering DNA with certain defined characteristics.

As suggested [45], *accumulation nodes* are added, to highlight the possible observations on DNA transfer:

- POI’s DNA is on the card;
- Unknown DNA is on the card.

Additionally, given that background DNA is a variable taken into consideration in our study, a *root node* considering the probability of recovering background DNA is added and it has a parental relationship with the finding node “Unknown DNA on the card”. When DNA from an unknown source is recovered, the presence of background DNA has to be included, because said unknown profile may be attributable to background DNA or an unknown, alternative offender (AO) [45]. Lastly, a *result node* is added. This node displays the possible outcomes and their posterior probability values, depending on which proposition is considered true (either Hp or Hd). When constructing the BN with software, an additional node, termed function node, can be added to calculate the ratio of said probabilities (and thus directly calculate the LRαs). Since the BN shown in Figure 3 was not constructed with software, a function node is not reported.

**Figure 4:**
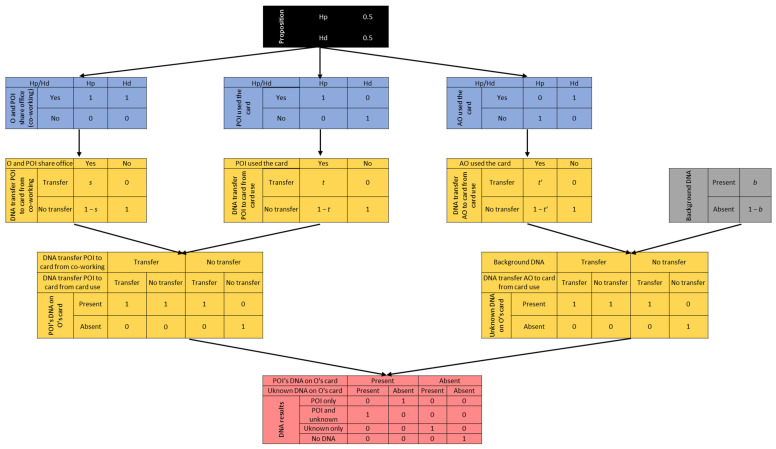
Bayesian Network constructed, displaying probability values for each node. *t*, *t’*, *s*, and *b* probabilities were informed by experimentation. To infer the probability of DNA transfer for both direct and secondary transfer mechanisms, two criteria were applied. The observation of a reportable profile (log10LRϕ > 6) has been labelled as the 1st level criterion. The 2nd level criterion is POI being the major contributor in terms of DNA amounts.

The probabilities underlying each node are shown in Figure 4. The probabilities of the sub-activity, accumulation, and results nodes are Boolean, meaning they either have a value Pr = 0 or Pr = 1, depending on the parental proposition considered to be true, and thus on the disputed activity. The only activity that is not being disputed and is accepted as true by both the prosecution and defence is the sharing of the workspace (thus the corresponding sub-activity node has Pr = 1 under both propositions) [36,48].

The probability of transfer events, either primary or secondary, and background DNA, are inferred from our observations and will inform the respective finding nodes and the background DNA root node. As of today, there is no general agreement on which DNA mixture attribute is the most suitable for comparing results from different transfer scenarios [16,49,65,69,70]. Therefore, to inform our probability values, POI’s relative contribution in ng to the trace (Table 3) was used as a quality indicator for the DNA trace’s properties we were investigating. However, since our study is explorative in nature, only a qualitative and discrete assessment was performed, meaning that only the presence or absence of the POI as a major contributor to the trace was taken into account. This reasoning is based both on our observations and on previous works according to which the order of subjects handling an object could at times be inferred by the most prominent contributor [18,21,24,71,72].

The probabilities, and their values, that will inform our network are the following:

- *t* = the probability that a DNA amount, leading to a reportable major contribution from POI, will be transferred from POI and recovered on the card, given that POI used the card.
- *t’* = the probability that a DNA amount, leading to a reportable major contribution from an unknown, alternative offender (AO), will be transferred from UNK and recovered on the card, given that UNK used the card.
- *s* = the probability that a DNA amount, leading to a reportable major contribution from POI, will be transferred and recovered on O’s card, given that O and POI share the same workspace, and O left the card on POI’s desk.
- *b* = probability of observing background DNA on the card.

To infer the probability of DNA transfer for both transfer mechanisms, two criteria based on the chosen discriminatory parameters (log10LRϕ and POI’s DNA amount) were applied. In particular, the formula “reportable major profile” verbally translates the probability of observing DNA traces with both a log10LRϕ > 6 and POI as a major contributor. For personal identification purposes, an “extremely strong support” in favour of the inclusion of an individual as a donor of a trace requires an LRϕ greater than 10^6^ [37,73,74]. Therefore, the observation of a reportable profile (log10LRϕ > 6) has been labelled as the 1st level criterion. The 2nd level criterion is POI being the major contributor in terms of DNA amounts.

*t* and *t’* are the probabilities relative to the primary transfer scenario, and they were extrapolated by determining what was the proportion of primary transfer events that presented a major reportable contribution from POI to the trace.

- 1st Level: probability of log10LRϕ > 6 = 9/11 = 0.818;
- 2nd Level: probability of POI being a major contributor (ng) = 7/9 = 0.778.

The probability of log10LRϕ > 6 **and** POI being a major contributor = 0.818 × 0.778 = 0.636.

*s* is the probability of observing POI as the reportable major contributor in the secondary transfer scenario.

- 1st Level: probability of log10LRϕ > 6 = 4/11 = 0.364;
- 2nd Level: probability of POI being a major contributor (ng) = 2/4 = 0.5.

The probability of log10LRϕ > 6 and POI being a major contributor = 0.364 × 0.5 = 0.182.

As for the background DNA probability, it is calculated as the number of events in which a third, unknown contributor, other than POI and O, was present in both direct and secondary transfer events, and this value is *b* = 19/22 = 0.864.

To evaluate the strength of evidence depending on activity level propositions, LRα was manually calculated under the four different possible outcomes of the DNA analysis, according to the formulae reported by Gill et al. in the Supplementary material [49]. The results are listed in Table 4. As can be observed, when POI only is recovered as the contributor to the trace, other than O (who is always assumed to be present), the prosecution’s proposition (that POI used the card) is ten times more likely than the defence proposition. When both POI and an unknown individual are observed, so the trace has three contributors, the LRα is slightly lower but still favours the prosecution. As can be expected, when no DNA other than that of the owner is retrieved, the LRα has a neutral value of 1 and when only unknown DNA is observed (and not POI’s), the LRα value favours the defence hypothesis.

Our results were then tested by creating the BN in the software Hugin Expert [75].

**Table 4:** Formulae used to calculate Likelihood Ratios (LRα), as indicated in Gill et al. [49].

DNA Analysis Outcome	Formulae Pr(E|Hp)/Pr(E|Hd)	LRα
POI only	(1−((1−*s*) (1−*t*))) (1−*b*)/s(1−*t’*) (1−*b*)	10.6003
POI and unknown	(1−((1−*s*) (1−*t*)))*b*/*s*(1−((1−*t’*) (1−*b*)))	3.507
Unknown only	(1−*s*) (1−*t*)*b*/(1−((1−*t’*) (1−*b*))) (1−*s*)	0.331
No DNA	(1−*s*) (1−*t*) (1−*b*)/(1−*s*) (1−*t’*) (1−*b*)	1

## 4. Discussion

### 4.1. Phase I–Shedder Status

As reported in previous studies, great inter- and intra-individual variations exist in the amount of DNA deposited through touch [3,9,11,12,15,17]. Additionally, other features that influence the deposition of touch DNA are the length of contact, the type of item and the nature of the surface, the presence of moisture on the surface or in the sample, etc. [62,63,76]. In terms of DNA quantities deposited on our surface of choice for the assessment of shedder status (the plastic tube), our results are comparable with those reported in the literature based on the same material, nature of the contact, and length of contact [3,22,62,63]. In particular, they were in line with the results obtained by Fonneløp et al. [65], who performed the same kind of assessment (with falcon tubes). As far as the participant’s individual characteristics, age and skin conditions seem to be factors determining the shedding propensity [4,10,11,12,13,14,15,16,17]; however, participants in the study fell in the age range between 25 and 65 years of age and had no known skin conditions, thus minimizing interindividual variability. To maintain realistic casework conditions, participants were allowed to perform routine daily activities, which, of course, varied among them.

Keeping this in mind, our experiment allowed for the broad classification of participants into shedding categories that are not dependent on strict laboratory experimental conditions. It has to be considered that the shedding propensity of an individual may vary depending on activities performed and intra-individual variability [9,41,42,77,78,79,80]. For the same reason, the profile completeness at time *t*0 after handwashing was included in the calculations for shedding status assessment. Handwashing reduces the quantity of retrievable DNA (whose quantity then increases over time) [13]; however, we considered this value important for a general assessment of shedding tendency, given different activities that an individual can perform before committing a criminal offence (or generally touching something). Additionally, despite our observations being too few, none of the participants showed an extremely low shedding value. We observed that 75% of participants (3 out of 4) appeared to belong to a more intermediate shedding range, thus informing our belief that a binary categorization system (Poor/High shedder) may be too simplistic, while also considering that individuals may fall at different points of a continuous distribution range [8,25,31,65,77]. Therefore, an Intermediate shedder category was added, even though we are well aware that this categorization has to be informed by more data.

### 4.2. Phase II—Direct and Secondary Transfer

This study aimed to investigate qualitative and quantitative characteristics of DNA traces given different transfer mechanisms, so as to verify whether, based on said attributes of a DNA mixture, it is possible to discriminate the mode of transfer [31]. In terms of qualitative observations, both evaluation approaches for O and POI’s profile completeness showed high mean values in the direct and secondary transfer alike; however, some differences are present. O’s profile completeness remains more or less constant for both direct and secondary transfer (Table 2, Figure 2D,F), since O used the card as per their usual habits prior to both experiments. Conversely, the degree of POI’s profile completeness, for both methods of calculation, differs between transfer mechanisms. POI’s profile completeness (Table 2, Figure 2E,G) in the direct transfer samples is higher than in the secondary ones. Given the secondary transfer of DNA by means of an intermediate surface, a lower quality of POI’s profile is to be expected. However, these results are to be interpreted considering that POI’s DNA was freshly deposited on the cards right before their sampling.

In terms of DNA quantity deposited and retrieved from credit cards, in both direct and secondary transfer, our results are generally comparable with those reported in other studies, albeit slightly lower [17,22,24,62,63,65,81]. However, the limited surface and length of use of a credit card have to be taken into account. Differences in DNA quantities can be observed between primary and secondary transfer: secondary transfer touch DNA samples show roughly half the DNA quantities as compared to direct transfer samples. Results are consistent with previous studies regarding touch DNA [17,22,62,63] also considering that, as common sense suggests, the cards directly handled, without gloves, by the POI will yield a higher DNA amount than the cards of the secondary transfer scenario. When considering the secondary transfer scenario, we expected a reasonably good outcome. Our expectations were based on the fact that secondary touch DNA transfer by means of an object (the desk surface) resulted in a greater amount of DNA being transferred when the trace was fresh, the surfaces involved were hard and non-porous (such as plastic), and slight pressure and friction was applied [15,16,62,63,76,82,83]. This could explain why, in 50% of secondary transfer DNA traces that returned an LRϕ > 10^6^, POI was the major contributor.

Breaking down the observed DNA quantities by contributor, the owner (O) is expected to always be present in the trace in the same quantity and quality, ideally due to the same frequency of use of the card prior to transfer experiments. However, despite presenting roughly the same profile completeness values both in the direct and secondary transfer, O contributes to the DNA trace with a slightly lower DNA amount in the secondary transfer scenario (Figure 2J). This could be due to the sterilization of the cards after the first experiment and to the fact that only one month passed between the first (direct transfer) and second experiment (secondary transfer). This period of time was initially considered to be sufficient for O to deposit DNA amounts reflective of frequent and extensive use of the card. However, in a future study, it would be useful to investigate how a longer time interval between experiments could change O’s DNA amounts deposited on the card. Additionally, O has a higher mixture proportion in the secondary transfer rather than in the primary one (Figure 2H), realistically due to POI’s decrease in contribution in terms of mixture proportion. In fact, for the direct transfer scenario, POI is generally a major contributor, both in mixture proportion and DNA amounts deposited, as well as showing a higher profile completeness degree (Figure 2E,G,I,K, “D” transfer). All of these three parameters drop in value for the secondary transfer (Figure 2E,G,I,K, “S” transfer). This difference between O and POI’s characteristics is reasonable given that, when directly handling the card, POI leaves fresher DNA and in higher quantities in relation to the DNA traces previously deposited, whose quantity and quality may have reduced over time. Additionally, this is in line with previous studies, where the last individual to handle an object was generally retrieved as the most prominent contributor [18,21,24,71,72].

### 4.3. Background DNA

As could be expected, the probability of recovering DNA originating from unknown sources is quite high. Our results are comparable with those described in the literature, which reports the presence of background DNA in more than 60% of the samples [15,18,19,20,21,22,23,24]. These observations highlight the importance of carrying out experiments that are as close to real life as possible since non-prevalent DNA is present virtually everywhere [41]. This consideration is particularly relevant for substrates and items that could be frequently subjected to foreign DNA transfer. Credit cards are often handled by non-owners, such as shop clerks, or may be used in an ATM withdrawal where other individuals’ cards are inserted thus leaving behind their DNA, that could be subsequently picked up by other cards. Therefore, the detection of unknown DNA from such an item is highly probable.

### 4.4. General Considerations

Despite the good preliminary results obtained, the LRα values show moderate to low statistical support in favour of Hp. In particular, the LRαs obtained are similar to results obtained in other touch DNA studies [42,49,51,65,70]. For the discussion of the results, the following considerations have to be kept in mind: the owner’s contribution is always assumed; both the prosecution and the defence agree on the possibility of POI’s DNA being transferred due to co-working with O; the card was handled bare-handed; if POI did not use the card, someone else (AO) did, given the evidence of cash withdrawal. For the “POI only” and “POI and unknown” outcomes, the LRα values indicate that the observed evidence is, respectively, 10.6 and 3.5 times more likely if the prosecution’s hypothesis on the mode of transfer (direct deposition) was true than if Hd was true (secondary transfer). The LRα for the “POI and unknown” scenario still favours Hp; however, it yields a lower LRα than the “POI only” outcome. In fact, the presence of a third, unknown contributor strengthens the defence’s hypothesis of an unknown individual (AO) being the offender and POI’s DNA on the card resulting from a secondary transfer due to co-working.

As expected, the “Unknown only” outcome is in favour of Hd, albeit with low statistical support, given the fact that the probability of recovering background DNA is quite high (*b* = 0.864) and that the origin of the unknown DNA found on the card is not known (from AO or background DNA). The “No DNA” scenario, meaning that only DNA from the owner is recovered, is not informative. The result is explained by the fact that the absence of DNA does not exclude that POI handled the card. Given that neither POI’s nor an unknown donor’s profiles have been observed, POI and AO have the same probability of leaving a detectable DNA amount leading to a reportable major contribution (*t* and *t’*, respectively) as much as they have the same probability of leaving no detectable DNA traces (1−*t* and 1−*t’*, respectively). In fact, in some instances “masked touching”, meaning touching an object without leaving detectable DNA, has been observed [28,30,66,72,84,85].

The present study offers the investigative and preliminary basis for the set-up of another, more in-depth study; therefore, performing a number of subsequent steps would be appropriate.

First, it would be advisable to increase the number of individuals taking part in the study. As already mentioned, the small dataset here investigated poses some limitations. Individuals’ shedding tendency should be further investigated in a larger population: more information regarding intra- and inter-individual variation has to be collected to support our preliminary classification of shedding status into three classes, while also taking into consideration variability due to performed activities. The supplementary data thus obtained on shedding status would be of useful implementation to the Bayesian Network here constructed.

Second, the number of experiments carried out for both primary and secondary transfer should be implemented. This can be achieved by increasing the number of individuals investigated, creating pairings that are higher in number and more varied in terms of shedding status differences between participants. This would allow us to obtain more information on POI’s prevalence in the DNA mixtures and on other qualitative and quantitative DNA profiles’ characteristics. Additionally, statistical simulations would allow increasing the number of samples based on the effective observations, thus increasing the number of statistically relevant events per DNA transfer mechanism.

Third, sensitivity testing should be carried out, to analyse how the LRα values would change depending on plausible ranges of probabilities for primary and secondary transfer, conditioned upon different values of POI’s relative contribution, not only based on presence/absence calculations [44,65]. For example, Fonneløp and colleagues [65] showed how a continuous quantitative evaluation of the chosen DNA attribute and sensitivity testing increased the value of the evidence.

Fourth, DNA persistence and recovery on credit cards should be investigated. To maximize DNA recovery rates, we decided to collect the samples right after the transfer event; however, it is commonly known that DNA trace quantities decrease as the time elapsed from the deposition of the traces increases [16,49,50]. Lastly, the complexity of the Bayesian Network could be improved by implementing other nodes regarding, for example, information on shedder status, DNA persistence and recovery (thus comparing different recovery techniques), and extraction efficiency.

## 5. Conclusions

This study aimed to investigate qualitative and quantitative characteristics of DNA traces given different transfer mechanisms, so as to verify whether, based on said attributes of a DNA mixture, it is possible to discriminate the mode of transfer. Based on a real-life casework scenario, direct and secondary DNA transfer experiments were set up, after investigating the DNA shedding propensity of the participants. Qualitative and quantitative differences were observed between the traces produced during the primary transfer scenario and the secondary transfer scenario. The DNA trace attribute herein taken into consideration is the relative mixture contribution in ng of POI and discrete observations (presence/absence of POI as a major contributor) were used to inform the probabilities of primary and secondary transfer events. Given the realistic experimental conditions, the objects utilized in the study (cards and desktops) were not sterilized and participants were not asked to wash their hands before handling the cards. Therefore, the majority of the samples showed the presence of a third, unknown contributor. This observation was used to infer the probability of observing background DNA. Based on the relevant case-specific information and the data collected, a Bayesian Network was constructed. Likelihood Ratios at activity level (LRα) were calculated for each of the possible outcomes resulting from the DNA analysis. In instances where only POI and POI plus an unknown individual are retrieved, the values obtained show moderate to low support in favour of the prosecution proposition. This means that observing POI as a contributor to the trace and their relative contribution higher than the owner’s, it is more likely if POI used the card than if POI’s DNA was indirectly transferred due to co-working with the card’s owner. These results are promising in terms of discriminating between traces resulting from a primary and secondary transfer, especially in a case that involves touch DNA with case circumstances that, to our knowledge, have not been previously investigated. A more in-depth analysis of the qualitative and quantitative characteristics of a touch DNA trace, of individual shedder status, and of the sensitivity should be the natural next steps in the development of this study.

## Figures and Tables

**Table 1:** Concentration values and profile completeness proportions for touch DNA samples deposited by participants at four time points after handwashing: *t*0 (immediately after), *t*1 (1 h), *t*2 (2 h), and *t*3 (3 h). Participants were asked to hold, for 30 s and with both hands, a 15 mL falcon tube.

Participant	Time Point	Concentration (ng/μL)		DNA Quantity (ng in 30 μL)		Profile Completeness		Shedder Category
		Values	Average	Values	Average	Values	Average	
A	*t*0	0.0011	>0.0029	0.033	>0.086	0.13	0.41	Intermediate
	*t*1	0.0032		0.096		0.22		
	*t*2	0.004		0.120		0.63		
	*t*3	0.0032		0.096		0.66		
B	*t*0	0.0006	0.0018	0.018	0.053	0	0.30	Poor
	*t*1	0.0016		0.048		0.34		
	*t*2	0.0021		0.063		0.34		
	*t*3	0.0028		0.084		0.53		
C	*t*0	0.0094	0.0191	0.282	0.572	0.59	0.75	High
	*t*1	0.0176		0.528		0.81		
	*t*2	0.0223		0.669		0.84		
	*t*3	0.0270		0.810		0.75		
D	*t*0	0.0005	0.0033	0.015	0.097	0.06	0.32	Intermediate
	*t*1	0.0016		0.048		0.13		
	*t*2	0.0038		0.114		0.51		
	*t*3	0.0070		0.210		0.56		

**Table 2:** DNA analysis results for the 11 direct transfer experiments and the 11 secondary transfer experiments. DNA quantities are reported both as a concentration and as entire DNA yield per sampled card (ng in 30 μL). Profile completeness was calculated employing a double approach, taking into consideration both the proportion of unique alleles of O and POI present in the trace and the proportion of all possible alleles of O and POI present in the trace.

Transfer Mechanism	Sample Code	Concentration (ng/μL)	DNA Quantity (ng in 30 μL)	Shared Alleles		Profile Completeness (Unique Alleles)		Profile Completeness (All Alleles)	
				O with POI	POI with O	O	POI	O	POI
Direct transfer	D1	0.012	0.345	5	5	0.63	0.78	0.69	0.81
	D2	0.071	2.13	5	5	0.67	0.96	0.72	0.97
	D3	0.061	1.836	10	8	0.77	1	0.84	1
	D4	0.034	1.026	8	10	0.89	0.93	0.91	0.94
	D5	0.065	1.944	5	5	0.96	0.96	0.97	0.97
	D6	0.074	2.226	10	8	1	1	1	1
	D7	0.023	0.687	8	8	0.54	0.63	0.63	0.69
	D8	0.031	0.942	8	8	0.58	0.58	0.59	0.63
	D9	0.078	2.343	5	5	0.85	1	0.88	1
	D10	0.056	1.692	5	5	0.63	1	0.69	1
	D11	0.069	2.07	5	5	0.93	1	0.94	1
Secondary transfer	S1	0.013	0.39	5	5	0.96	0.24	0.88	0.31
	S2	0.025	0.753	5	5	0.84	0.64	0.81	0.72
	S3	0.018	0.537	10	8	0.77	0.63	0.81	0.69
	S4	0.026	0.783	8	10	0.92	0.77	0.94	0.84
	S5	0.036	1.071	8	8	0.82	0.96	0.84	0.97
	S6	0.057	1.701	10	8	1	0.68	1	0.78
	S7	0.006	0.171	8	8	0.33	0.44	0.38	0.47
	S8	0.023	0.675	5	5	0.74	0.96	0.75	0.97
	S9	0.016	0.468	5	5	0.93	0.93	0.91	0.88
	S10	0.017	0.504	5	5	0.85	0.78	0.91	0.78
	S11	0.006	0.174	5	5	0.22	0.41	0.31	0.5

**Table 3:** DNA analysis results for the 11 direct transfer experiments and the 11 secondary transfer experiments. Statistical evaluation has been carried out using EuroForMix v 4.0.1. LR at sub-source level (LRϕ) has been calculated with the continuous model, and the mixtures proportions reported for O, POI, and unknown are calculated by EuroForMix as well. O, POI, and Unknown’s contribution to the trace in terms of DNA quantity (ng) was calculated by multiplying the contributors’ relative mixture proportion with the DNA quantity (ng in 30 μL) of the trace.

Transfer Mechanism	Sample Code	Number of Contributors	log10LRϕ	Mixture Proportion			Contribution in ng		
				O	POI	Unknown	O	POI	Unknown
Direct transfer	D1	3	12.36	0.49	0.35	0.16	0.170	0.119	0.057
	D2	3	14.29	0.19	0.57	0.24	0.405	1.214	0.511
	D3	3	21.13	0.72	0.16	0.12	1.322	0.294	0.220
	D4	3	12.84	0.33	0.49	0.19	0.334	0.502	0.190
	D5	3	14.88	0.37	0.48	0.15	0.719	0.933	0.292
	D6	3	15	0.40	0.45	0.15	0.890	0.979	0.334
	D7	3	5.08	0.40	0.36	0.24	0.247	0.275	0.165
	D8	3	2.76	0.39	0.29	0.32	0.363	0.269	0.309
	D9	3	22.58	0.14	0.77	0.09	0.328	1.804	0.209
	D10	3	20.71	0.09	0.73	0.18	0.154	1.235	0.305
	D11	3	22.63	0.16	0.81	0.03	0.331	1.677	0.056
Secondary transfer	S1	2	1.30	0.12	0.88	/	0.046	0.344	/
	S2	3	5.55	0.48	0.32	0.20	0.361	0.241	0.151
	S3	3	1.06	0.27	0.24	0.49	0.145	0.129	0.263
	S4	3	−0.17	0.57	0.14	0.29	0.446	0.110	0.227
	S5	3	10.14	0.27	0.46	0.27	0.289	0.493	0.289
	S6	3	7.03	0.78	0.14	0.08	1.327	0.238	0.136
	S7	2	6.48	0.38	0.62	/	0.066	0.106	/
	S8	3	7.24	0.46	0.17	0.37	0.322	0.115	0.250
	S9	3	3.99	0.39	0.29	0.32	0.183	0.136	0.150
	S10	3	4.45	0.45	0.30	0.25	0.227	0.151	0.126
	S11	2	5.79	0.29	0.71	/	0.051	0.124	/

## Data Availability

Data is available upon request to the corresponding author.
